# Novel FAP-Targeted Heptamethine Cyanines for NIRF Imaging Applications

**DOI:** 10.1021/acs.molpharmaceut.4c01232

**Published:** 2025-02-15

**Authors:** Rebecca Rizzo, Martina Capozza, Laura Conti, Lidia Avalle, Valeria Poli, Enzo Terreno

**Affiliations:** † Department of Molecular Biotechnology and Health Sciences, University of Turin, Piazza Nizza 44/bis, Turin 10126, Italy; ‡ Department of Molecular Biotechnology and Health Sciences, University of Turin, Via Nizza 52, Turin 10126, Italy; § DISIT, University of Eastern Piedmont, Viale Teresa Michel 11, Alessandria 15121, Italy

**Keywords:** FAP, near-infrared fluorescence imaging, breast cancer, heptamethine cyanines, optical imaging

## Abstract

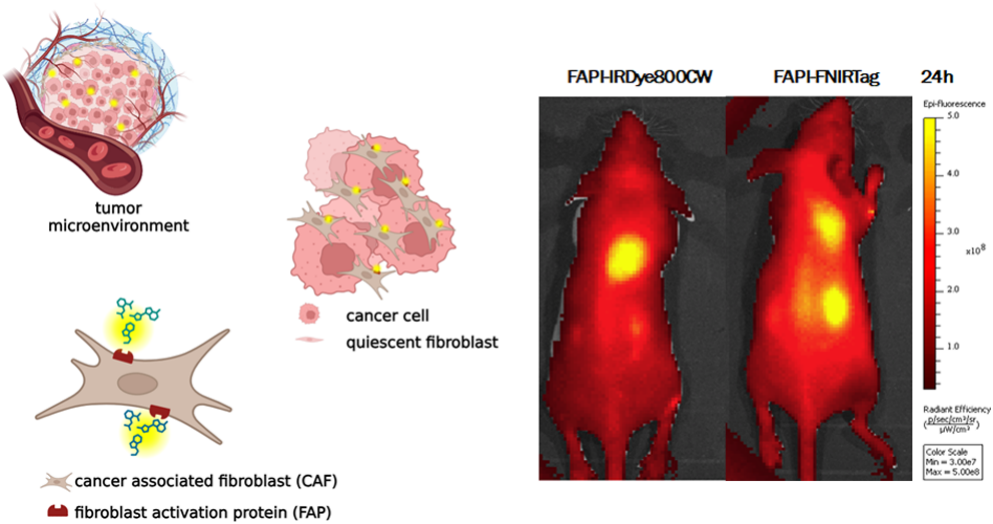

Fibroblast activation protein (FAP) is a pan-cancer target that is useful for imaging, ideally all epithelial cancers. This work aimed to develop, characterize, and validate two novel FAP-targeted probes for optical imaging, both in *vitro* and *in vivo*. IRDye800CW and FNIRTag heptamethine cyanines were conjugated to the NH precursor of the well-known FAP inhibitor FAPI-46, which is widely employed in nuclear medicine. In addition to synthesis, the dyes were characterized in terms of physicochemical properties, biodistribution, and imaging performances in a breast cancer tumor model. FAPI-FNIRTag showed a stronger fluorescence and higher photostability compared to FAPI-IRDye800CW. Notably, both compounds exhibited strong tumor accumulation in TUBO breast cancer-bearing mice 24 h postadministration, suggesting potential for further investigation as fluorescence-guided surgery (FGS) agents.

## Introduction

Fibroblast activation protein (FAP) is a leading theranostic biomarker because of its crucial role in angiogenesis, growth, and progression in cancer.^1^ FAP is a transmembrane serine protease overexpressed by cancer-associated fibroblasts (CAFs) in over 90% of epithelial cancers,^2^ including breast,^3,4^ colon,^4,5^ and pancreatic^4,6^ tumors. Thus, FAP has been considered as a pan-cancer target in several preclinical approaches and clinical trials. Considering that, the development of diagnostic approaches by targeting FAP would be of great importance. Optical imaging is a noninvasive technique based on the interaction of light with tissues that guides the clinician’s decision-making in real-time. For instance, in surgery, intraoperative fluorescence imaging represents a key player in improving cancer surgical outcomes.^7^ Surgical intervention remains a primary treatment for patients with solid tumors, but incomplete resections or unnecessary removal of healthy tissues can significantly affect the prognosis of patients. In breast cancers, for example, many of which are nonpalpable, margin positivity rates range from 5 to 49%.^8^ Indeed, among the factors that influence local recurrence, margin resection is one of the few modifiable risk factors, which prompts surgeons to seek new approaches to reduce positive surgical margin (PSM) rates.^9^ Tumor-targeted, near-infrared fluorescent (NIRF) imaging is an emerging field of real-time intraoperative cancer imaging based on tumor-targeted NIRF dyes that have become relevant in breast cancer, as demonstrated by literature studies primarily in phases 1 and 2.^10^ Besides undeniable advantages such as the noninvasive nature, the immediate response during surgery intervention, the tunability of probes to obtain functional and molecular information, and the use of nonionizing radiation, optical imaging is affected by some criticisms. Among these, fluorophore conjugation often alters the properties of both the fluorophore and the molecule to which it is conjugated.^11^ Specifically, important parameters such as brightness, target binding, *in vivo* stability, and pharmacokinetics (PK) are often impacted.^12^ Thus, the development of novel tumor-targeted NIRF dyes has increased interest in the fluorescence-guided surgery (FGS) field. To identify a pan-tumor marker for use in the imaging of solid tumors, some research groups have investigated FAP-targeting ligands for fluorescence imaging. Nevertheless, some suffer from unwanted accumulation in healthy tissues, while others exhibit short tumor retention times.^13,14^ More recently, Mukkamala and co-workers^15^ have successfully developed the FAP-targeted S0456 dye as capable of imaging most solid tumors (human triple-negative breast cancer, human colorectal cancer, human glioblastoma, human head and neck cancer, human pancreatic cancer, murine breast cancer, and human nasopharyngeal cancer), demonstrating the notable relevance of FAP imaging in the optical imaging field. To further exploit this need, we developed two novel fluorescent probes based on the FAPI-46 structure, one of the most promising FAP inhibitors showing nanomolar affinity for FAP and high tumor-to-background ratio in positron emission tomography (PET) imaging.^16^ The conjugated fluorophores of this work are IRDye800CW and FNIRTag due to their different charges (IRDye800CW has a net charge of −3, while FNIRTag has a zwitterionic structure) and structure peculiarities. IRDye800CW has a polyanionic structure and, although widely used in clinical trials, suffers from nonspecific *in vivo* binding, which prevents the maximization of fluorescence intensity at the target site.^17^ FNIRTag has a zwitterionic structure, which may improve its tumor-to-background ratio.^18^ On these premises, this work aims to synthesize, validate, and compare, both *in vitro* and *in vivo*, two novel FAP-targeting fluorescent probes for NIRF imaging applications.

## Experimental Section

### Chemicals

All chemicals were obtained from Sigma-Aldrich. All solvents for Ultra-performance liquid chromatography–mass spectrometry (UPLC-MS) analysis and the purification procedure were purchased from VWR. Activated NHS-ester cyanines (FNIRTag-NHS, IRDye800CW-NHS) were obtained from Bio-Techne.

### Instrumentation

Electrospray ionization-mass spectrometry (ESI-MS) analyses were performed on a Waters system (3100 Mass Detector, 2525 Quaternary Pump, 2767 Sample Manager, 2996 PDA detector). Purification procedures were conducted on an AKTA Purifier high-performance liquid chromatography (HPLC) System. Ultraviolet/visible (UV/vis) spectrophotometric measurements were conducted on a UV/vis spectrophotometer (6715, Jenway). Fluorescence measurements were performed on a FluoroMax-4 Spectrofluorometer (Horiba Scientific).

### Cell Cultures and In Vitro Experiments

Seronorm (human serum) was purchased from Sero AS. Human serum albumin was purchased from Sigma-Aldrich. Mammary carcinoma (lobular carcinoma that arose spontaneously in a BALB-neuT mouse) TUBO cells were kindly provided by Professor Laura Conti’s research group. HEK293-FAP cells, stably expressing FAP, were generated by Dr. Lidia Avalle. The pCMV6-Kan/Neo-mFAP expression vector, encoding murine FAP, was purchased from Origene (cat. MC206606) and used to transfect HEK293 cells with Lipofectamine (Invitrogen, cat. no. 11668019). Transfected cells were then selected with 500 μg/mL Geneticin (G-418, Gibco cat. 10131035) for 7 days. In parallel, HEK293 cells transfected with a construct lacking the FAP coding sequence were generated as negative controls. FAP expression was measured by Western Blot using antibodies against FAP (Santa Cruz, sc-100528) and β actin as a loading control (Santa Cruz, sc-4778).

### Animals

Balb/c female mice and athymic nude mice were purchased from Envigo. Mice were maintained under sterile and pathogen-free conditions at the animal facility of the Molecular Biotechnology Center and treated in accordance with EU and institutional guidelines, with the approval of the Animal Care and Use Committee of the University of Turin and the Italian Ministry of Health (*n*° 298/2022-PR).

### Synthesis and Purification

The piperazine precursor of FAPI-46, previously synthesized according to an already published procedure,^16^ was conjugated via an amide bond with the *N*-hydroxysuccinimidyl ester of IRDye800CW or FNIRTag fluorescent molecules. The reactions were conducted as follows: 1 equiv of a fluorophore NHS-ester (2 mg) was dissolved in 0.5 mL of dimethylformamide (DMF) in a 2 mL cylindrical flask, followed by a dropwise addition of 2.1 equiv of FAPI-46 (amine precursor) premixed with 6 equiv of DIPEA, and stirred at room temperature for 3 h in the dark. After evaporating the solvent under reduced pressure, 500 μL of purified water was added to the crude reaction mixture, and the solution was loaded into the preparative chromatographic column. For the detailed purification procedures, see the Supporting Information. The two synthesized molecules were obtained in the form of a green powder.

### UV–Vis, Fluorescence Measurements, and Fluorescence Phantom Imaging

To quantify the obtained compounds, three different dilutions of each molecule were prepared in phosphate-buffered saline (PBS), and the relative absorbance values were measured. The Lambert–Beer equation was used to calculate the concentration of each solution, using the molar coefficient of the unconjugated fluorophore (ε = 2 × 10^5^ cm^–1^ M^–1^ FNIRTag and 2.4 × 10^5^ cm^–1^ M^–1^ IRDye800CW),^19^ upon the assumption that the conjugation of each fluorophore to the NH-derivative of FAPI-46 does not affect the ε value of the fluorophores. The optical properties of these conjugates were analyzed by spectrophotometric and fluorometric analysis. Absorbance spectra were recorded at different concentrations in both PBS pH 7.4 and PBS:MeOH (1:1). Fluorescence emission and excitation spectra were recorded at different concentrations in both PBS at pH 7 4 and PBS/MeOH (1:1). Fluorescence emission spectra were recorded using the excitation wavelengths of the unconjugated fluorophores.^19^ Phantom imaging of FAPI-IRDye800CW and FAPI-FNIRTag was performed in a 96-multiwell plate (0–500 nM) by employing an excitation filter of 740 nm and an emission filter of 790 nm (IVIS, PerkinElmer). Fluorescence signals in average radiant efficiency [p/s/cm^2^/sr]/[μW/cm^2^] were plotted as a function of the dye’s concentration (6.25–500 nM).

### Stability

Chemical stability was assessed by UPLC-MS measurements. Fluorescent compounds were incubated in the human serum at 37 °C. After selected incubation times (0, 2, 6, 24, and 48 h), acetonitrile was added to precipitate protein. Centrifugation was performed at 16,060 rcf for 10 min, and the supernatant was analyzed by UPLC-MS. The final concentration of the fluorescent compound was set to 5 μM. UPLC-MS measurement of the serum was performed to account for the contribution of the serum to the MS signal. Photostability was determined by irradiating the compounds for 2 h with a 200 W xenon lamp at 60 s intervals (120 points). Photobleaching was evaluated by measuring the decrease in fluorescence intensity and plotting the normalized fluorescence intensity (emission values divided by the initial fluorescence for each compound) as a function of irradiation time.

### Albumin Binding

Serum protein binding measurements were performed using human serum albumin (≥97% purity, Sigma-Aldrich). The dyes were incubated at a fixed concentration of 0.15 μM with increasing concentrations of albumin (0–30 μM). Incubation was conducted in PBS at room temperature for 1 h. Afterward, the fluorescence emission profile was recorded by employing the same protocol as described previously for fluorescence measurements. The normalized fluorescence emission at the maximum value was plotted against the albumin concentration. The data were fitted with the equation (*y* = *F*_bound_ × (F_bound_ – F_free_) + F_free_ × [dye]), from which the binding dissociation constant and the fluorescence of the bound dye were derived.

### Cell Lines

HEK293-FAP-transfected cells were maintained in DMEM (1 g/L glucose) supplemented with 10% fetal bovine serum and 2 μg/mL puromycin. HEK293 cells were maintained in DMEM (1 g/L glucose) supplemented with 10% fetal bovine serum. TUBO cell line was maintained in DMEM supplemented with 20% fetal bovine serum. Cells were cultured in a humidified incubator with 5% CO_2_ at 37 °C.

### Animal Studies

For the *ex vivo* assessment of FAP expression, 5 week-old female Balb/c mice (*n* = 5) were subcutaneously injected in the right shoulder with 10^5^ TUBO cells suspended in 100 μL of PBS. Tumor growth was checked twice a week by a caliper, and tumor tissue was excised when the tumor size reached 400 mm^3^. For *in vivo* imaging (*n* = 4/group) and biodistribution studies (*n* = 3/group), 5-week-old female athymic mice were subcutaneously injected in the right shoulder with 10^5^ TUBO cells suspended in 100 μL of PBS. Five nanomoles of FAPI-IRDye800CW or FAPI-FNIRTag was intravenously injected into the tail vein, and fluorescence imaging (IVIS Spectrum, PerkinElmer) was performed at different time points (0, 1, 2, 4, 6, 24, 48, 72, 96, 168 h p.i.). For biodistribution studies, 5 nmol of the dyes were intravenously injected into the tail vein. The mice were euthanized by cervical dislocation 24 h p.i., the main organs were excised, and the quantification of the signal was performed by measuring the average radiance efficiency by the IVIS Spectrum. ROI (region of interest) analysis was performed to obtain a fluorescence quantification.

### Flow Cytometry

For uptake experiments, 4 × 10^5^ HEK293-FAP cells were incubated with the fluorescent compounds (FAPI-IRDye800CW or FAPI-FNIRTag) at a final concentration of 0.5 μM or with rat anti-mouse FAP antibody (MAB9727 - Bio-Techne) at a final concentration of 0.25 μg/10^6^ cells. Donkey anti-rat IgG (H + L) highly cross-adsorbed secondary antibody, Alexa Fluor 488-conjugated, was used as the secondary antibody (1:2000). For competition experiments, unconjugated NH-FAPI-46 was incubated with cells for 10 min prior to the addition of conjugated dyes (100 ×, 50 μM). The HEK293 cell line was employed as the negative control. The samples, after the addition of propidium iodide (PI) to assess cell viability, were analyzed on a BD-FACSVerse instrument (BD, New Jersey) using a 488 nm laser with a 530/40 nm emission filter and a 633 nm laser with a 750-long pass emission filter.

To assess FAP expression in tumor tissues, single-cell suspensions were prepared from the fresh primary tumors. MAB9727 was used as anti-FAP antibody (0,25 μg/10^6^ cells), and A21208 Donkey anti-rat IgG (H + L) highly cross-adsorbed Alexa Fluor 488 was used as secondary antibody (1:1000). After *ex vivo* biodistribution experiments were conducted, the FAPI-IRDye800CW and FAPI-FNIRTag dyes were employed as fluorescent probes to assess FAP expression in TUBO tumor tissues. Samples were acquired on BD-FACSVerse (BD Bioscience) and analyzed with FlowJo10.5.3, as stated above.

### Immunohistochemistry

To assess FAP expression, tumor tissues were incubated overnight at 4 °C with anti-FAP antibody (ab207178) at a 1:100 dilution. Goat anti-rabbit IgG H&L (HRP) (ab97051) was incubated for 1 h at room temperature at a 1:200 dilution. Images were acquired using aDM6 Leica microscope at 4×, 10×, and 20× magnification.

### Statistical Analysis

The Student’s *t*-test was used to compare the differences between the two groups. A *p*-value of <0.05 was considered statistically significant. Graph preparation and statistical analysis were performed using GraphPad Prism version 8.

## Results and Discussion

### Synthesis, UV–Vis, and Fluorescence Measurements

**Figure 1:**
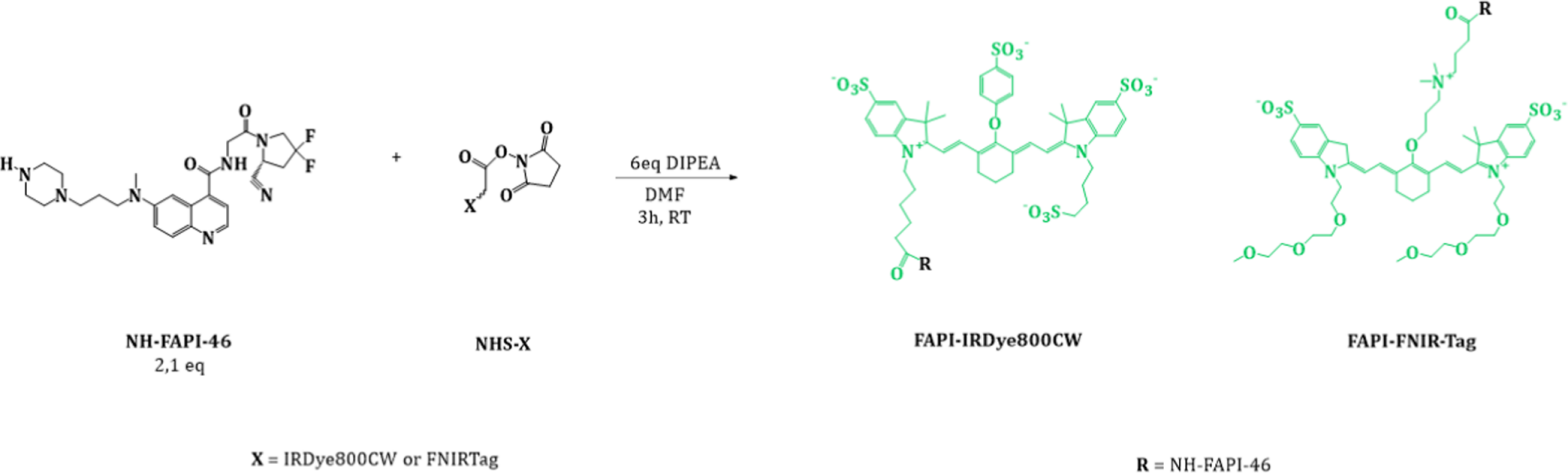
Synthetic protocol for FAPI-IRDye800CW and FAPI-FNIRTag.

FAPI-IRDye800CW and FAPI-FNIRTag were obtained by following the synthetic protocol reported in Figure 1. The purity of compounds (>97%) was assessed by integrating the peaks observed at 254 and 700 nm (Figures S1 and S2). The yields of FAPI-IRDye800CW and FAPI-FNIRTag were 29 and 18%, respectively.

**Figure 2:**
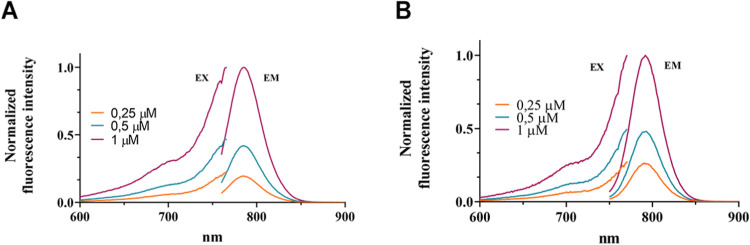
Fluorescence excitation and emission spectra. Fluorescence excitation and emission spectra in PBS (pH = 7.4) of (A) FAPI-FNIRTag (λ_ex_ = 740 nm, λ_em_ = 788 nm) and (B) FAPI-IRDye800CW (λ_ex_ = 730 nm, λ_em_ = 790 nm).

**Figure 3:**
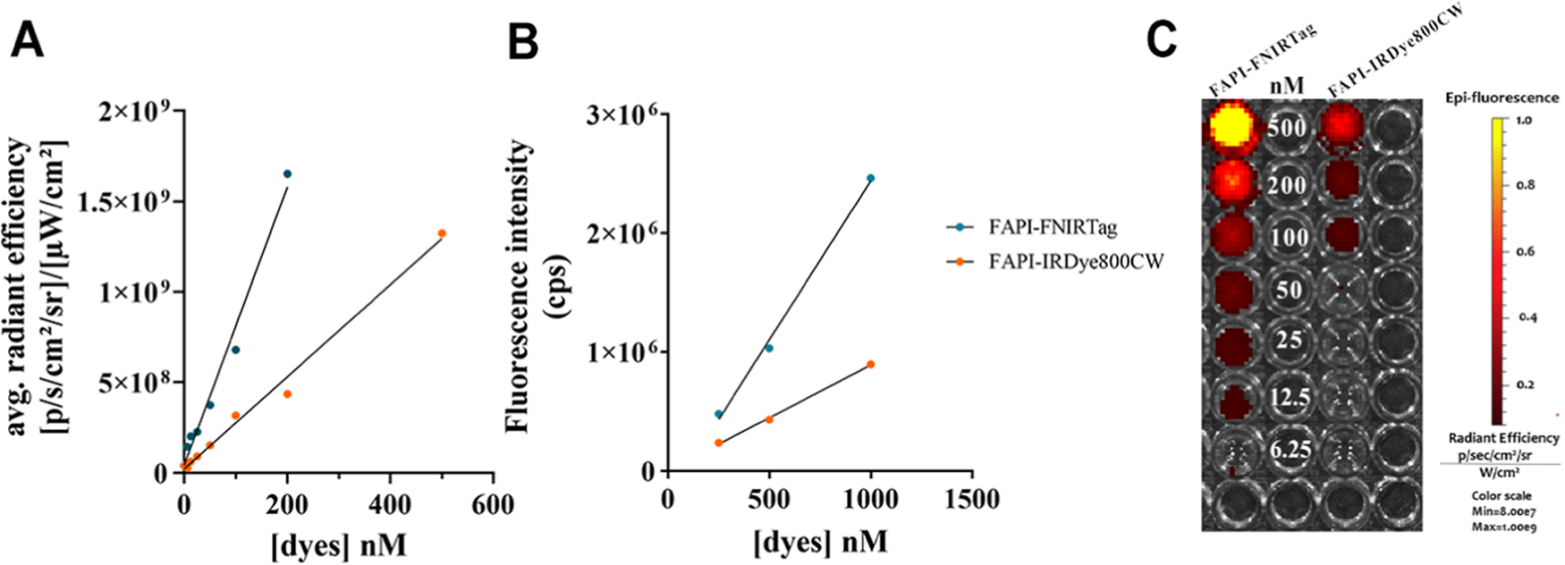
Fluorescence linearity and phantom imaging. (A) Average radiance efficiency comparison between FAPI-FNIRTag and FAPI-IRDye800CW at different concentrations (IVIS measurements and phantom imaging). (B) Fluorescence emission comparison (cps) between FAPI-FNIRTag and FAPI-IRDye800CW based on fluorimeter measurements (λ_ex_ = 740 nm for FAPI-FNIRTag, λ_ex_ = 730 nm for FAPI-IRDye800CW). (C) Phantom imaging comparison between FAPI-FNIRTag (on the left) and FAPI-IRDye800CW (on the right) (λ_ex_ = 740 nm, λ_em_ = 790 nm).

The photophysical properties of the two synthesized compounds were investigated. Since conjugation of a fluorophore can often lead to modification of its properties,^20^ especially in terms of aggregation or quantum yield, two different media, PBS (50 mM, pH = 7.4) and (PBS)/MeOH (1:1), were used. Absorbance measurements were used to quantify each product, using molar extinction coefficients known from the literature for the unconjugated dye, under the assumption that the ε values of the dyes were not affected by the conjugation. As depicted in Figure 2, the emission spectra revealed a higher fluorescence for FAPI-FNIRTag than FAPI-IRDye800CW (Figure 3B). Moreover, the absorbance values were found to be higher in PBS/MeOH (Figure S3) for both dyes, and this may be due to the action of methanol on breaking the hydrogen bonds present in not fluorescent aggregates, as previously shown by Luciano et al.,^19^ or due to a change in the chemical environment. As for the excitation spectra (Figures S4 and S5), however, it is possible to observe, as already shown by Luciano et al.,^19^ the presence of a hypsochromic band (shifted toward blue) at around 700 nm, probably related to the formation of small H-aggregates. The H-aggregates represent the most common aggregates for heptamethine cyanine dye molecules due to the face-to-face stacking.^21^

To further investigate the optical behavior of the products, the ratio of the hypsochromic band to the monomer band was calculated for both solvents as the concentration of the dyes increased.^22,23^ The ratio was almost unaffected by the concentration, thus suggesting that no aggregation occurred in the investigated concentration range (0–1 μM). Similar experiments carried out on the NIRF scanner (Figure 3C) confirmed the linear correlation between fluorescence and concentration (0–500 nM) and the higher fluorescence emitted for FAPI-FNIRTag, as reported in Figure 3A. Moreover, the ratio between the FAPI-FNIRTag and the FAPI-IRDye800CW fluorescence intensities was found to be comparable using the two instruments and dye stock solutions. In both cases, FAPI-FNIRTag showed a 3-fold enhancement in fluorescence intensity if compared to FAPI-IRDye800CW. This is not surprising since the development of FNIRTag by Luciano and co-workers aimed to enhance the brightness of bioconjugates thanks to chemical structure modification at the C4′ position.

### Photo- and Chemical Stability

**Figure 4:**
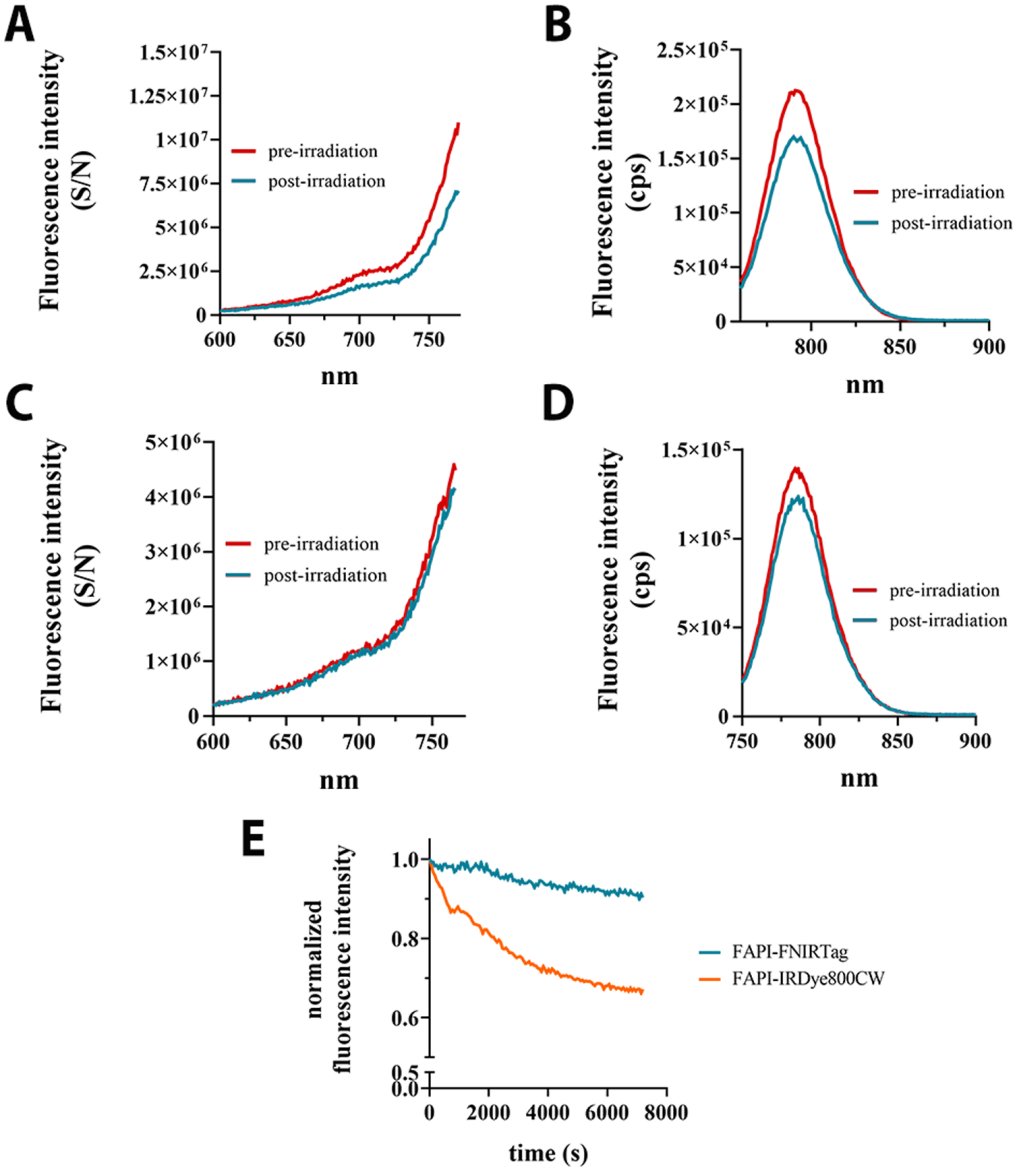
Photostability. (A) FAPI-IRDye800CW excitation spectra before and after irradiation. (B) FAPI-IRDye800CW emission spectra before and after irradiation. (C) FAPI-FNIRTag excitation spectra before and after irradiation. (D) FAPI-FNIRTag emission spectra before and after irradiation. (E) The time course of normalized fluorescence intensity decreases after irradiation (2 h, every 60 s) for FAPI-FNIRTag and FAPI-IRDye800CW.

Photostability is a crucial parameter for assessing the potential of an NIRF imaging probe involving several related phenomena. The photobleaching of a heptamethine cyanine dye is primarily caused by a bimolecular reaction between the polyene and photogenerated singlet oxygen (forming a dioxetane intermediate).^24^ Moreover, a recent study conducted by Nani and co-workers demonstrated that only exogenously generated ^1^O_2_, and no other common reactive oxygen species (ROS), is capable of replicating this reaction pathway and that regioselective cleavage at only two positions of the polyene, C_2_/C_1_′ and C_2_′/C_3_′, can be interpreted as being a consequence of the overall energetics of the dioxetane intermediates.^25^ Hence, to monitor the decrease in the fluorescence emission during irradiation, measurements were taken by recording the emission under repeated excitation. The data obtained were normalized to the initial fluorescence intensity. To check whether light irradiation causes changes in the excitation or emission spectra, both spectra were recorded before and after irradiation. As shown in Figure 4A–E, the two dyes exhibited different responses to light stimulation. FAPI-FNIRTag preserved 90% of its initial fluorescence, whereas FAPI-IRDye800CW experienced significant photobleaching early in the irradiation (15% after 49 min), approaching 60% by the end of the experiment. The latter observation could be due to a reduced singlet oxygen quantum yield for FNIRTag compared to IRDye800CW.

Chemical stability in serum was checked because it reported the possible covalent substitution of the 4′-phenoxy group by biological nucleophiles to give undesired dye degradation products.^17^ However, UPLC-MS measurements (Figures S6 and S7 in the Supporting Information) did not show any peaks corresponding to degradation products from C–O bond cleavage, as previously reported in the literature.^26^ Certainly, this finding does not ensure the same behavior *in vivo*, where this possibility must be carefully evaluated, particularly for FGS applications. Although two peaks were observed, they correspond to intact compounds, as confirmed by the identical SRM transition. The composition of the serum (ionic strength, pH) might have altered the chromatographic retention, leading to the formation of a double peak.^27^

### Albumin Binding

**Figure 5:**
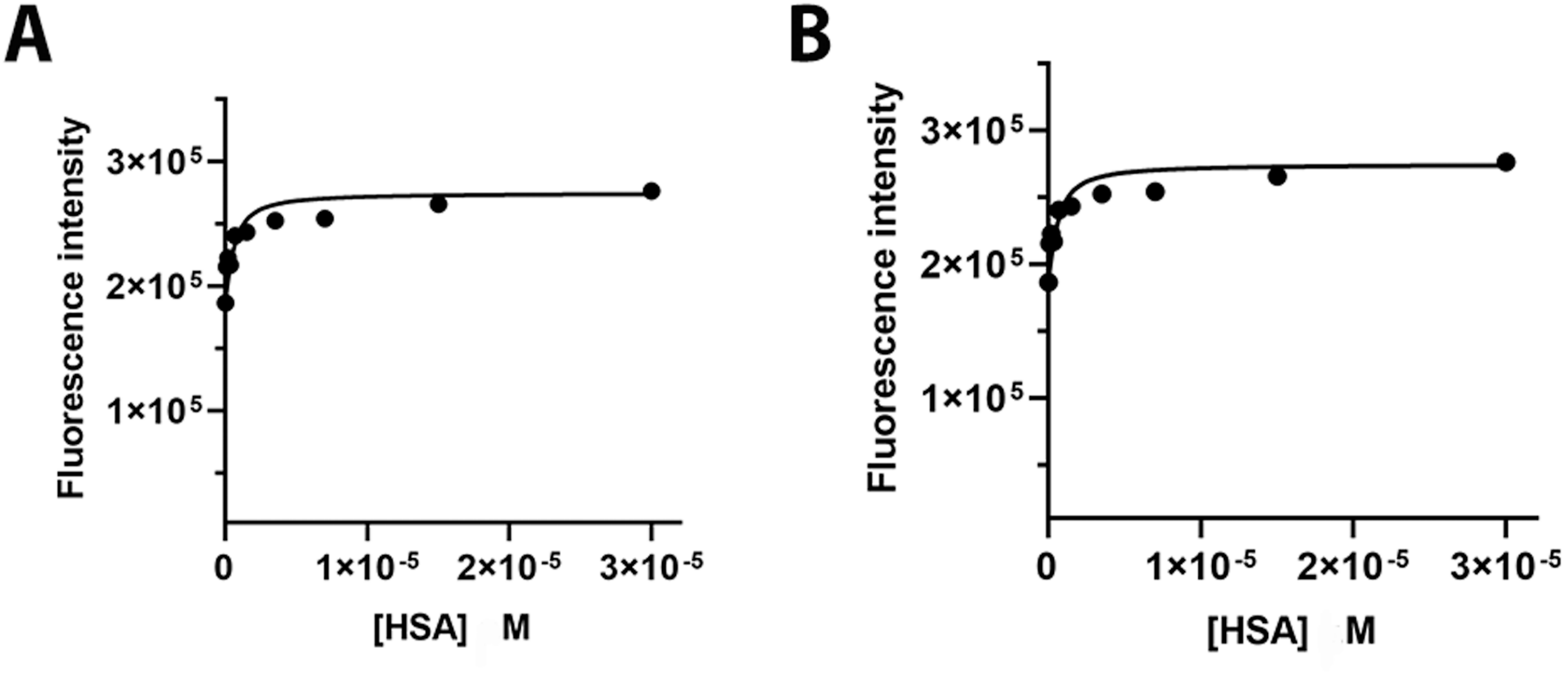
Serum albumin binding. Albumin binding data after 1 h incubation ([dye] = 0.15 μM, PBS pH 7.4) at RT. (A) FAPI-FNIRTag. (B) FAPI-IRDye800CW.

Albumin binding is an important parameter for predicting the biodistribution of pharmaceuticals, and it may affect the *in vivo* target specificity. Figure 5 shows the relationship between the fluorescence intensity and albumin concentration for the two dyes. From the observed values, it can be inferred that FAPI-IRDye800CW underwent a higher fluorescence enhancement (48 *vs* 27%) following albumin binding. However, by fitting the data to an appropriate binding isotherm equation, similar and low *K*_D_ constants were obtained (2.33 × 10^–7^ for FAPI-FNIRTag and 3.69 × 10^–7^ for FAPI-IRDye800CW), thus indicating that both compounds have a strong affinity for the protein.

### In Vitro Uptake on FAP-Expressing Cells

**Figure 6:**
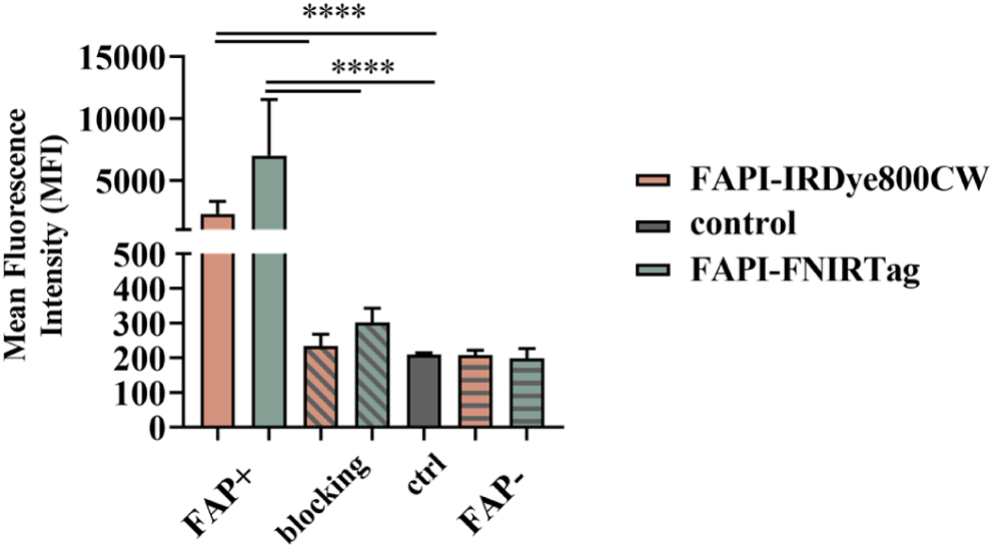
Fluorescent dye uptake on FAP-expressing cells. Fluorescence emission on FAP-expressing cells of dyes incubated at 0.5 μM (monochromatic histograms), dyes together with 100× excess of competitor (unconjugated FAPI-46) (angled patterned histograms), without any dyes (gray), and dyes on no-FAP-expressing cells (horizontal patterned histograms). Dot plots of acquired representative samples are fully reported in the Supporting Information. Data were shown as means ± SD **** *p* < 0.0001 (*n* = 3) *t*-test (α = 0.05).

To assess the *in vitro* uptake of the probes, FAP-transfected cells were employed, with the empty vector (the same cell line without the genetic insert) serving as the control. FAP expression was quantified by Western Blot (see Figure S8). Figure 6 shows the flow cytometry results, expressed as the mean fluorescence intensity (MFI), along with the corresponding histograms. Dot plots can be found in the Supporting Information (Figures S9 and S10). FAPI-IRDye800CW and FAPI-FNIRTag showed similar MFI values that were significantly higher than those of the control (FAP-expressing cells without any dyes), indicating a high uptake of both the dyes on FAP-expressing cells. To confirm the target specificity, a blocking experiment was conducted. The comparable MFI values between the blocked samples and the control demonstrated the specificity of the interaction between the dyes and the FAP protein. As further confirmation, the fluorescence measured on FAP-negative cell lines was equal to the control.

### FAP Assessment on TUBO Tumor Specimens by Flow Cytometry

**Figure 7:**
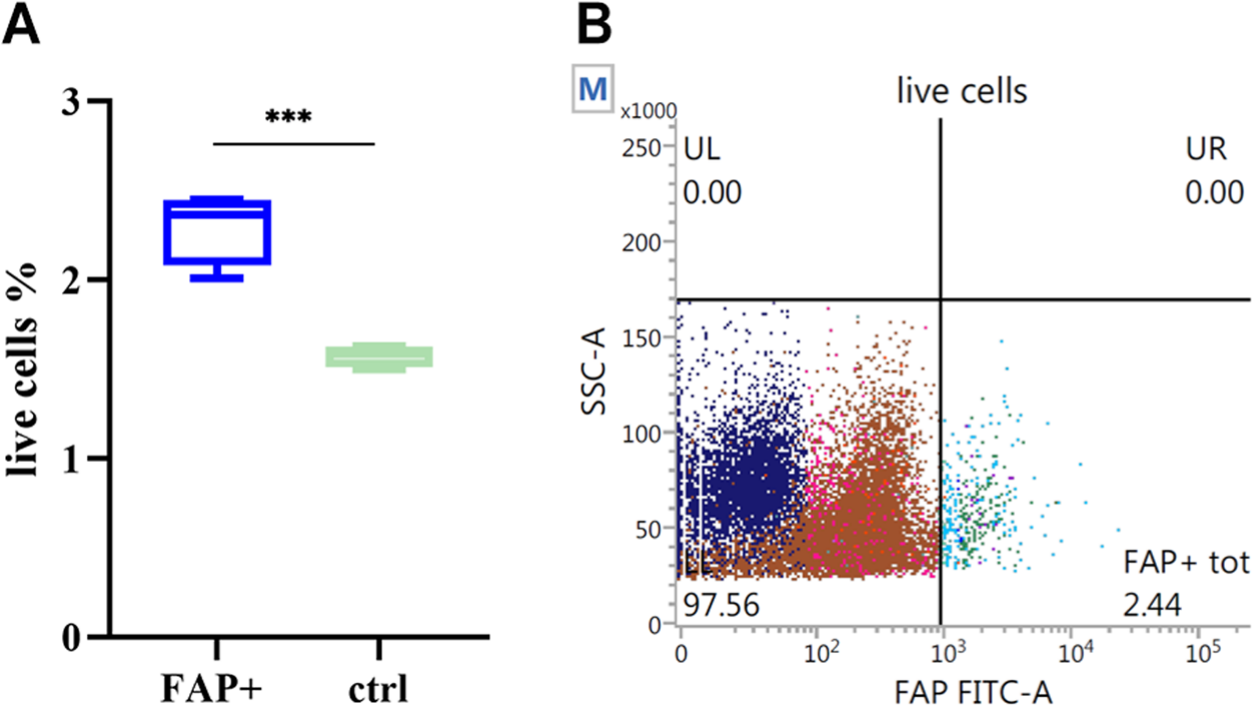
Flow cytometry results of FAP-expressing cells in TUBO tumors. (A) Percentage of FAP-expressing cells in live cells extracted from TUBO tumors in Balb/c mice. Control results refer to unstained FAP-expressing cells. Data were shown as means ± SD *** *p* = 0.0005 (*n* = 4) *t*-test (α = 0,05). (B) Dot plot of the TUBO tumor sample (BalB/c mice), gating on live cells.

Preliminary investigations of FAP expression of TUBO tumor specimens were addressed by flow cytometry measurements and immunohistochemical staining. Characterization of cell populations and individuation of FAP-positive cell subpopulations represent a key strategy to deeply understand the crosstalk between FAP and the tumor microenvironment, with beneficial implications for understanding FAP clinical significance. It is commonly known that breast cancer cells exhibit intratumoral heterogeneity, and emerging evidence points toward breast cancer CAFs being equally heterogeneous. It has been shown that human breast tumors contain at least four CAF subpopulations,^28^ indicating that the heterogeneity of breast cancer CAFs is not limited to experimental cancer models. Venning and co-workers looked at the temporal composition of CAF subpopulations during the development of an orthotopic breast tumor by implanting syngeneic 4T1 or 4T07 cells in Balb/c mice.^29^ Based on these examples, the % of FAP (on the live cells) in breast cancer tumor models (4T1, 4T07) settles down at around 1.5–2%.^29,30^ After digestion of the TUBO tumor specimen (see the Supporting Information for detailed protocol) and antibody incubation, FACS analysis was performed on cell pellets to quantify FAP. As depicted in Figure 7A, the percentage of live cells stained for FAP is equal to 2.3%, in line with the literature data. Figure 7B reports a representative dot plot of the FAP+ cell subpopulation.

### Immunohistochemistry

**Figure 8:**
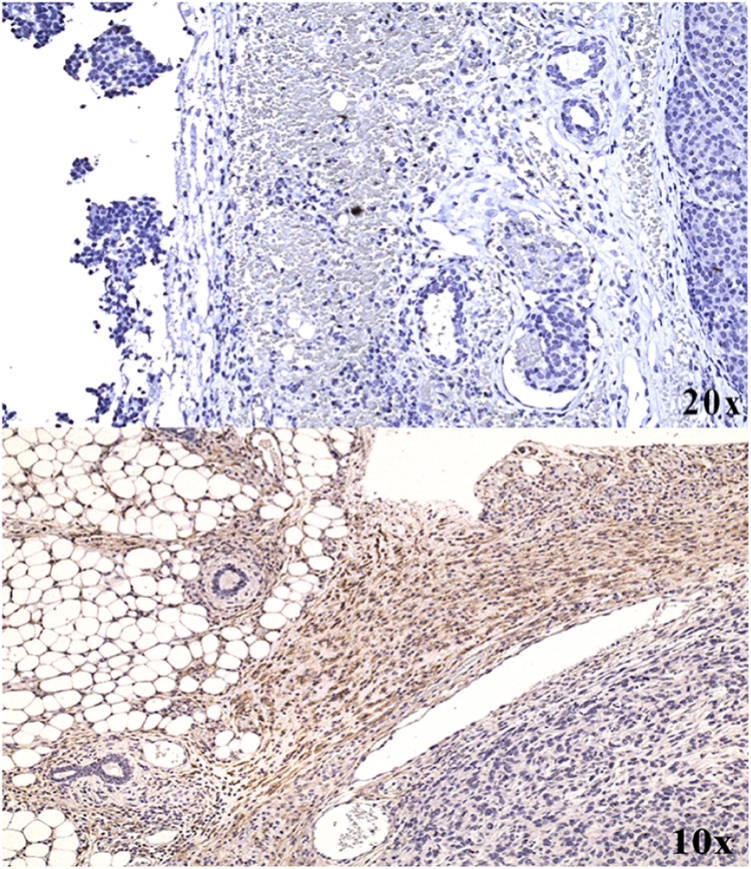
Immunohistochemical evaluation of excised tumor tissues from TUBO tumor-bearing Balb/C mice. Brown signals correspond to anti-FAP staining; nuclei were counterstained with hematoxylin. Immunohistochemical staining of excised tumor tissue in the absence of primary antibody anti-FAP above and immunohistochemical staining of excised tumor tissue (anti-FAP primary antibody) below.

FAP expression was further verified by means of immunohistochemical staining of the tumor tissues. Figure 8 reports a representative stained tumor tissue in which it is possible to appreciate the brown signal (DAB, 3,3′-diaminobenzidine), which shows the FAP-positive areas.

### In Vivo NIRF Imaging and Biodistribution Studies

**Figure 9:**
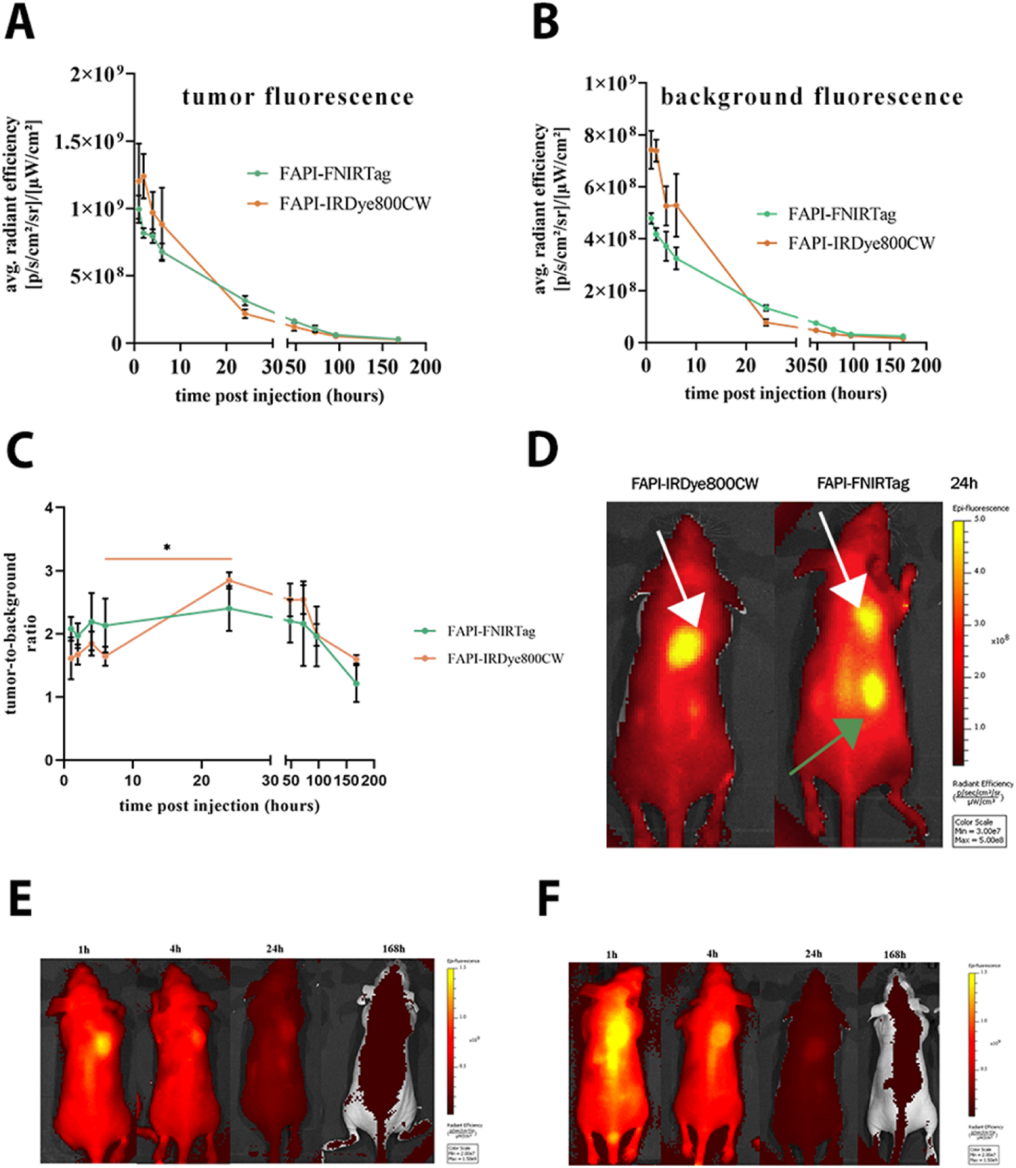
Fluorescence imaging. (A) Kinetics of i*n vivo* average radiance efficiency found for tumor tissue and (B) muscle (background). (C) Tumor-to-background ratio. * *p* = 0.0297 *t*-test (α = 0.05) (*n* = 3). (D) *In vivo* fluorescence imaging of TUBO tumor-bearing mice intravenously injected with 5 nmol of FAPI-IRDye800CW and FAPI-FNIRTag 24 h-postinjection (white arrows indicate the tumors; green arrow indicates the kidneys). (E) *In vivo* fluorescence imaging of TUBO tumor-bearing mice intravenously injected with 5 nmol of FAPI-FNIRTag at different time points. (F) *In vivo* fluorescence imaging of TUBO tumor-bearing mice intravenously injected with 5 nmol of FAPI-IRDye800CW at different time points.

**Figure 10:**
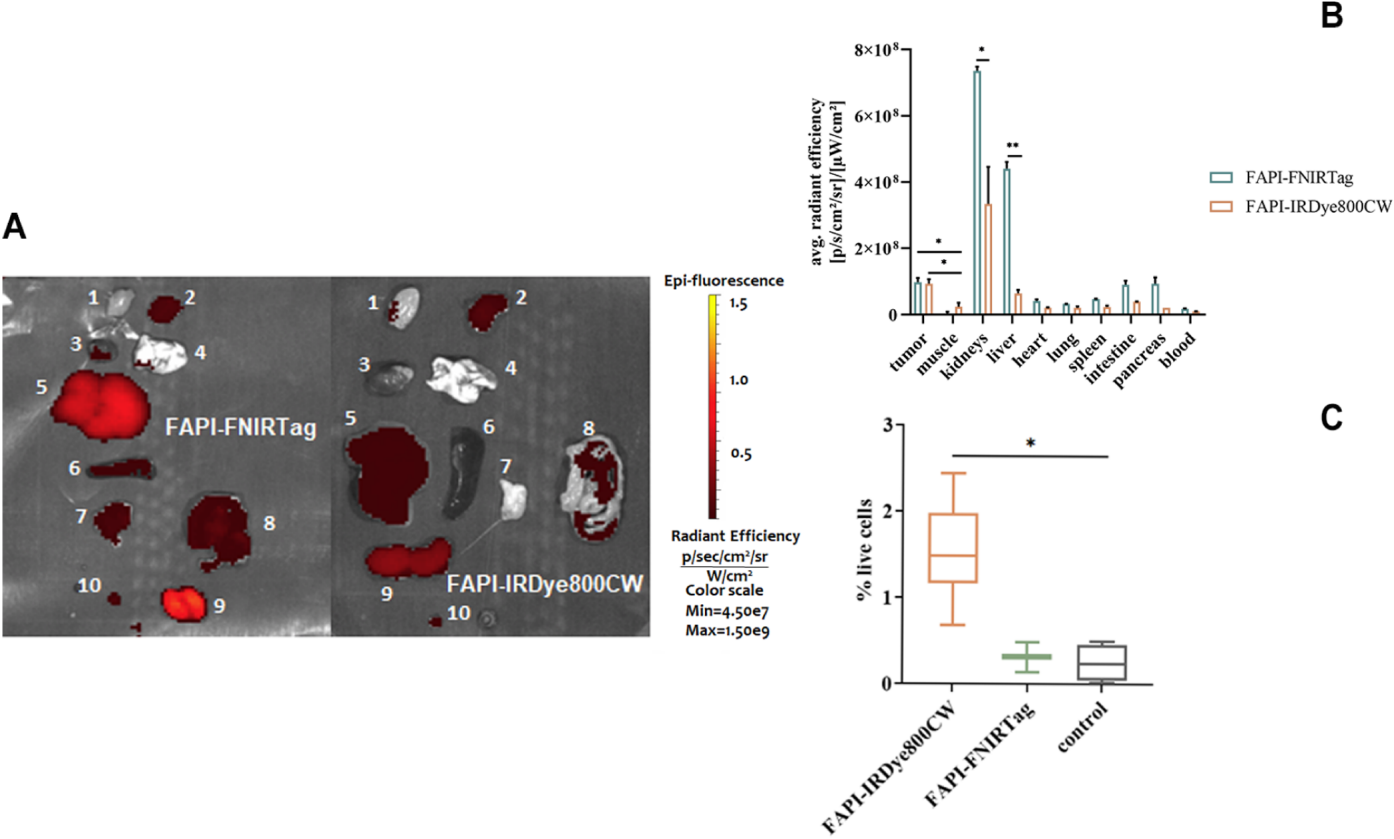
*Ex vivo* biodistribution study. (A) *Ex vivo* fluorescence imaging of the main organs excised from TUBO tumor-bearing mice *i.v.* injected with 5 nmol of FAPI-FNIRTag or FAPI-IRDye800CW (1: muscle, 2: tumor, 3: heart, 4: lung, 5: liver, 6: spleen, 7: pancreas, 8: intestine, 9: kidneys, 10: blood). (B) Biodistribution for FAPI-FNIRTag and FAPI-IRDye800CW (5 nmol) in TUBO tumor-bearing athymic mice 24 h postinjection. *p* = 0.0161 for FAPI-FNIRTag tumor/muscle; *p* = 0.0127 for FAPI-IRDye800CW tumor/muscle; *p* = 0.0030 for kidneys comparison; and *p* = 0.0014 for liver comparison. (*t*-test α = 0.05). (C) Percentage of dye-bound cells in live cells extracted from TUBO tumors in nude mice excised 24 h after *i.v.* injection of 5 nmol of FAPI-IRDye800CW or FAPI-FNIRTag. *p* = 0.024 (*t*-test α = 0.05).

*In vivo* FAP imaging at the preclinical level has typically been explored using animal models generated with FAP-transfected cell lines (mainly HT-1080-FAP, fibrosarcoma cell lines) or a few other models, including glioblastoma (U87) and breast cancer (4T1), but not limited to these. In this work, we aimed to explore the *in vivo* imaging application of two NIRF FAP-targeting probes for breast cancer imaging purposes by employing the HER2^+^ TUBO cell line. To the best of our knowledge, this is the first approach to image this type of preclinical model by means of FAP targeting, justifying the preliminary immunohistochemical evaluation and flow cytometry measurements on the tumor specimen. After the intravenous injection of 5 nmol of FAPI-IRDye800CW or FAPI-FNIRTag, NIRF imaging scans were acquired at different time points, and ROI analysis was performed on tumor and muscle (background reference) to quantify the average radiance efficiency. Figure 9 reports the kinetics of tumor (A) and muscle retention (B) within 7 days. Tumor-to-background (TBR) values were calculated at each time point (Figure 9C). The results obtained did not allow for confirmation of an effective tumor FAP-targeting for FAPI-FNIRTag dye, given the not-statistically significant difference among any time-point pair within 24 h. In contrast, a modest increment in the TBR was observed from 6 to 24 h for FAPI-IRDye800CW. In Figure 9D, the fluorescence images acquired 24 h postinjection for both agents are reported (white arrows indicate the tumors, green arrow indicates the kidneys). The series of NIRF images reported in Figure 9E,9F provide an overview of the temporal evolution during the 7 day investigation. To quantify the fluorescence in the main organs and to better understand the elimination kinetics, biodistribution studies were performed 24 h postinjection of 5 nmoles of the FAPI-conjugated dyes in TUBO tumor-bearing athymic nude mice. After the mice were euthanized by cervical dislocation, the main organs (tumor, kidneys, liver, heart, lung, pancreas, intestine, spleen) were excised, and the fluorescence signal was measured using the NIRF scanner. Figure 10A depicts the *ex vivo* fluorescence images of the excised organs, with their average radiance efficiency reported in Figure 10B. The data highlighted clear liver and kidney uptake for both dyes, with FAPI-FNIRTag showing a higher fluorescence in most of the organs (except tumor) compared to FAPI-IRDye800CW. As this dye showed *in vitro* a 3-fold higher fluorescence than FAPI-IRDye800CW (see Figure 3), this observation appears consistent with the view that the two dyes share a similar biodistribution. Dye biodistribution was further investigated by quantifying the fluorescence in the tumors excised 24 h postinjection. The results showed a modest difference between the FAPI-IRDye800CW fluorescence signal and the control signal, confirming an FAP-mediated uptake on the tumor. Differently, no statistically significant differences were observed between the FAPI-FNIRTag fluorescence signal and control (Figure 10C). These results seem to highlight a more specific binding of the IRDye800-conjugate compared to FNIRTag. Indeed, FACS allows isolation of the FAP-bound dye content, discarding the contribution from the tumor vasculature that is present both in the *in vivo* and *ex vivo* fluorescence signal.

FAPI-IRDye800CW and FAPI-FNIRTag represent two novel potential NIRF probes that take advantage of the ubiquitous FAP presence in solid tumors. FAPI-FNIRTag showed a higher relative fluorescence. Both dyes were found to specifically recognize FAP *in vitro*, suggesting robust potential as NIRF agents for optical imaging applications. However, several parameters contribute to the success of a novel fluorescent molecule as a contrast agent. Among them, serum protein binding, biodistribution, and consequent dye bioavailability represent the leading factors. Optical *in vivo* imaging showed different TBR dynamics for the two developed dyes, suggesting a FAP-mediated binding for FAPI-IRDye800CW.

## Conclusions

The implementation of diagnostic and intraoperative procedures to overcome the current limitations is still a matter of interest in oncology. Certain types of cancer, including breast cancer, suffer from high recurrence rates due to incomplete tumor resection. The development of new imaging probes and the investigation of new stromal biomarkers represent key strategies to explore. This work aimed to develop two novel NIRF imaging probes targeting fibroblast activation protein FAP for breast cancer management. The two developed NIRF agents were characterized optically, *in vitro* on FAP-expressing cells and *in vivo* in an unexplored FAP-expressing preclinical tumor model of breast cancer. The conjugation of both fluorophores with the FAP inhibitor FAPI-46 met the expectations regarding their fluorescence performances, as the higher fluorescence emission reported in the literature for FNIRTag was confirmed by our data. The good *in vitro* uptake of the dyes by FAP-expressing cells demonstrated that the conjugation with the FAPI-46-like inhibitor did not impact FAP specificity. Regarding albumin binding and *in vivo* imaging studies, the results were not as expected. The development of FNIRTag was based on the premise that IRDye800CW leads to higher nonspecific signals *in vivo*, partly due to its increased albumin binding. The albumin binding results did not display differences between the two probes in terms of binding strength, and the obtained results *in vivo* did not indicate the FNIRTag-conjugate as the best performing one in terms of higher TBR, as initially conceivable. From the *in vivo* fluorescence imaging results, it can be affirmed that FAPI-FNIRTag seems to lack clear FAP-targeting behavior, as confirmed by *ex vivo* FAP-quantification using flow cytometry on TUBO tumor specimens. FAPI-IRDye800CW exhibited an increase in TBR between 6 and 24 h postinjection, suggesting a potential application as an NIRF agent in this time range. Moreover, these results are even more promising if we consider that FAPI-IRDye800CW is a small molecule and that similar studies usually involve antibody-based agents. However, our study may be strengthened and expanded to find a possible time point between 6 and 24 h with a higher tumor uptake. With FAP being mainly employed in nuclear medicine as a target for PET agents, further strategies to consider may involve a different FAP-inhibitor targeting moiety or a different FAP-targeting approach, such as a peptide to improve the tumor-to-background ratio for fluorescence imaging applications.

## Data Availability

The data supporting this article have been included as part of the Supporting Information.

## Supporting Information Available

UPLC-UV/vis-MS preparative and analytical methods (Tables S1–S3); ESI-MS spectrum and the UPLC-UV/vis spectrum at 700 and 254 nm of FAPI-FNIRTag and FAPI-IRDye800CW, respectively (Figures S1 and S2); absorbance spectra of FAPI-FNIRTag and FAPI-IRDye800CW (Figure S3); fluorescence emission spectra and fluorescence excitation spectra of FAPI-FNIRTag and FAPI-IRDye800CW, respectively (Figures S4 and S5); UPLC-UV/vis spectra at 700 nm of FAPI-FNIRTag and FAPI-IRDye800CW to follow serum stability (Figures S6 and S7); FAP expression quantification in HEK-293FAP+ cells by WB analysis (Figure S8); and flow cytometry results for uptake experiments on FAP-expressing cells as dot plots for FAPI-FNIRTag and FAPI-IRDye800CW, respectively (Figures S9 and S10) (PDF)

## Notes

**Ethical statement** The study is reported in accordance with ARRIVE guidelines.
