# Supplementary material for: Novel FAP-Targeted Heptamethine Cyanines for NIRF Imaging Applications
Source: Mol Pharm. 2025 Feb 15;22(3):1518–28. doi: 10.1021/acs.molpharmaceut.4c01232 (PMC11881144; doi:10.1021/acs.molpharmaceut.4c01232)
Supplement: Supplementary file 1 — mp4c01232_si_001.pdf [file mp4c01232_si_001.pdf]

Novel FAP-targeted heptamethine-cyanines for NIRF imaging applications

Rebecca Rizzo[1], Martina Capozza[1], Laura Conti[1], Lidia Avalle[3], Valeria Poli[2], Enzo Terreno[1*]

[1] Department of Molecular Biotechnology and Health Sciences, University of Turin, Piazza Nizza 44/bis, Turin, 10126, Italy

[2] Department of Molecular Biotechnology and Health Sciences, University of Turin, Via Nizza 52, Turin, 10126, Italy

[3] DISIT, University of Eastern Piedmont, Viale Teresa Michel 11, Alessandria, 15121, Italy

*Email: enzo.terreno@unito.it

Supplementary Information

UPLC-UV/Vis-MS

The chromatographic runs were performed using an Acquity UPLC BEH C18 (130Å, 1.7 µm, 2.1 mm x 50 mm) column. The column oven was maintained at 25˚C and the elution solvents were water/trifluoroacetic acid 0.1% (solvent A) and acetonitrile/trifluoroacetic acid 0.1% (solvent B) for FNIR-Tag and ammonium acetate 7mM (solvent A) and acetonitrile/trifluoroacetic acid 0.1% (solvent B) for IRDye800CW. The two different gradients were listed below in Table S1.

FAPI-IRDye800CW

t (min)	%A	%B
0	90	10
12	50	50
14	0	100

FAPI-FNIRTag

t (min)	%A	%B
0	95	5
11	60	40
14	0	100

Table S1 Gradients used for reversed-phase analytical runs of NH-FAPI-46 fluorescent conjugates.

The flow rate was 0.4 mL/min. The high-resolution Waters 3100 Mass Detector was operated with an electrospray ion source in positive ion mode with a cone voltage of 20V. The intervals of mass scan were 50–1250 m/z. MS ions found were list below in Table S2. The chromatograms were reported in Figure S1.

	FAPI-IRDye800CW	FAPI-FNIRTag
$[M-H]^+$	-	-
$[M-2H]^{2+}$	742.75	771.85
$[M-3H]^{3+}$	-	514.95

Table S2 MS ions found for synthetized products.

UV/Vis spectra was recorded at 214 and 700 nm. The reported UV/Vis spectra (Figure S1) was recorded at 700 nm. The preparative chromatographic runs were performed on a AKTA Purifier System. XBridge Prep BEH300 column was used for IRDye800CW (A=ammonium acetate 7mM, B=CH_3CN) and FNIR-Tag conjugates purification (A=H_2O+TFA 0.1%, B=CH_3CN); the two different gradients were listed below in Table S3.

FAPI-IRDye800CW

t (min)	%A	%B
0	90	10
13	50	50
14	0	100

FAPI-FNIRTag

t (min)	%A	%B	
0	90	10	
2	90	10	
9	72	28	
15	72	28	
18	0	100	

Table S3 Gradients used for reversed-phase preparative runs.

A

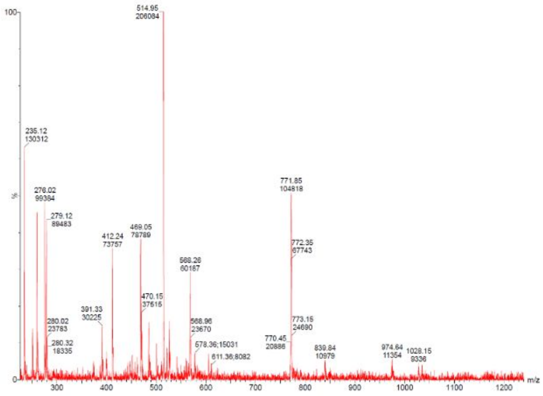

B

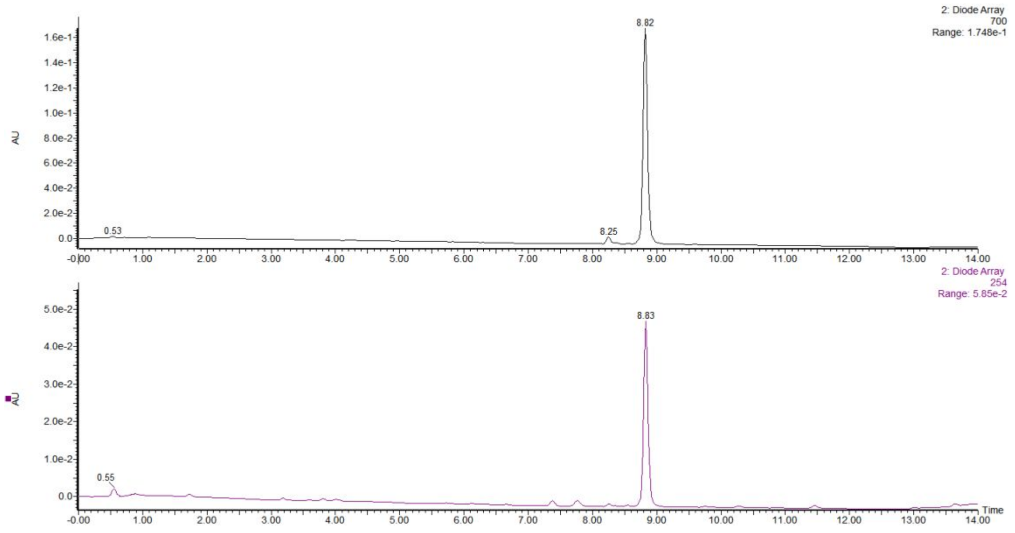

Figure S1 **(A)** ESI-MS spectrum of FAPI-FNIRTag, $[M-2H]^{2+}$ = 771.85 m/z, $[M-3H]^{3+}$ = 514.95 **(B)** UPLC-UV/Vis spectrum at 700 nm (purity > 97 %) and 254 nm (purity% > 97 %) of FAPI-FNIRTag.

A

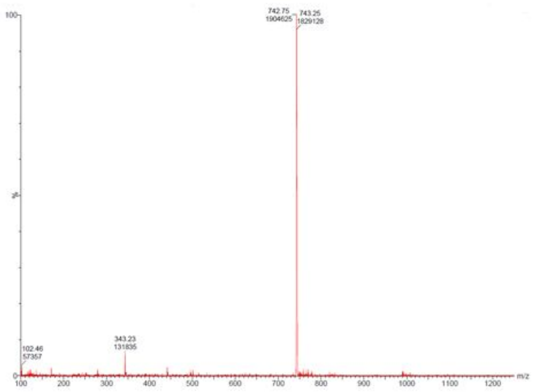

B

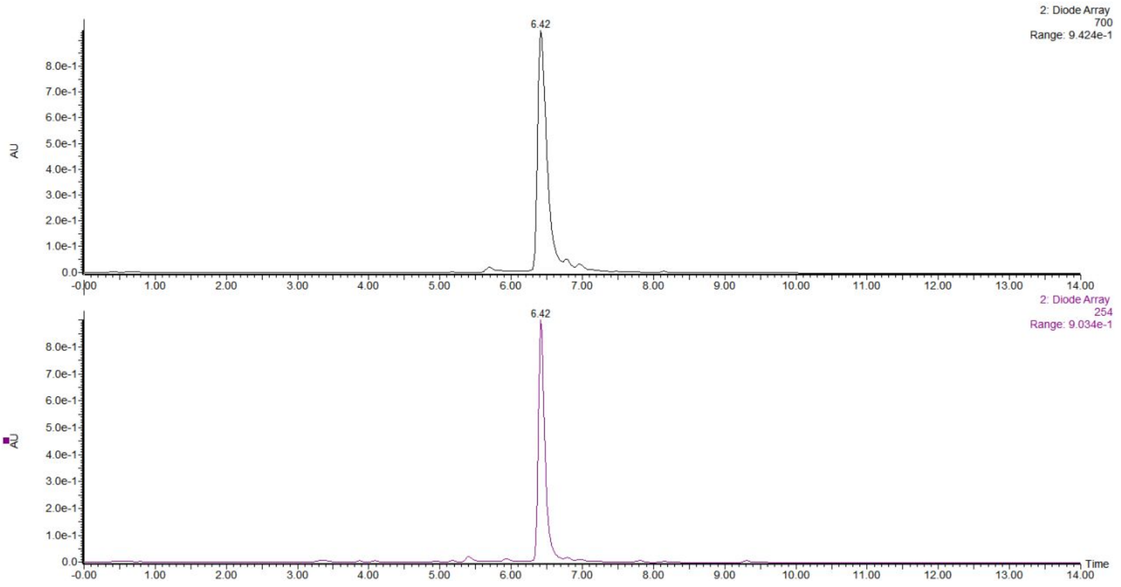

Figure S2 (A) ESI-MS spectrum of FAPI-IRDye800CW, $[M-2H]^{2+}$= 742.75 m/z. (B) UPLC-UV/Vis spectrum at 700 nm (purity > 97 %) and 254 nm (purity% > 97%) of FAPI-IRDye800CW.

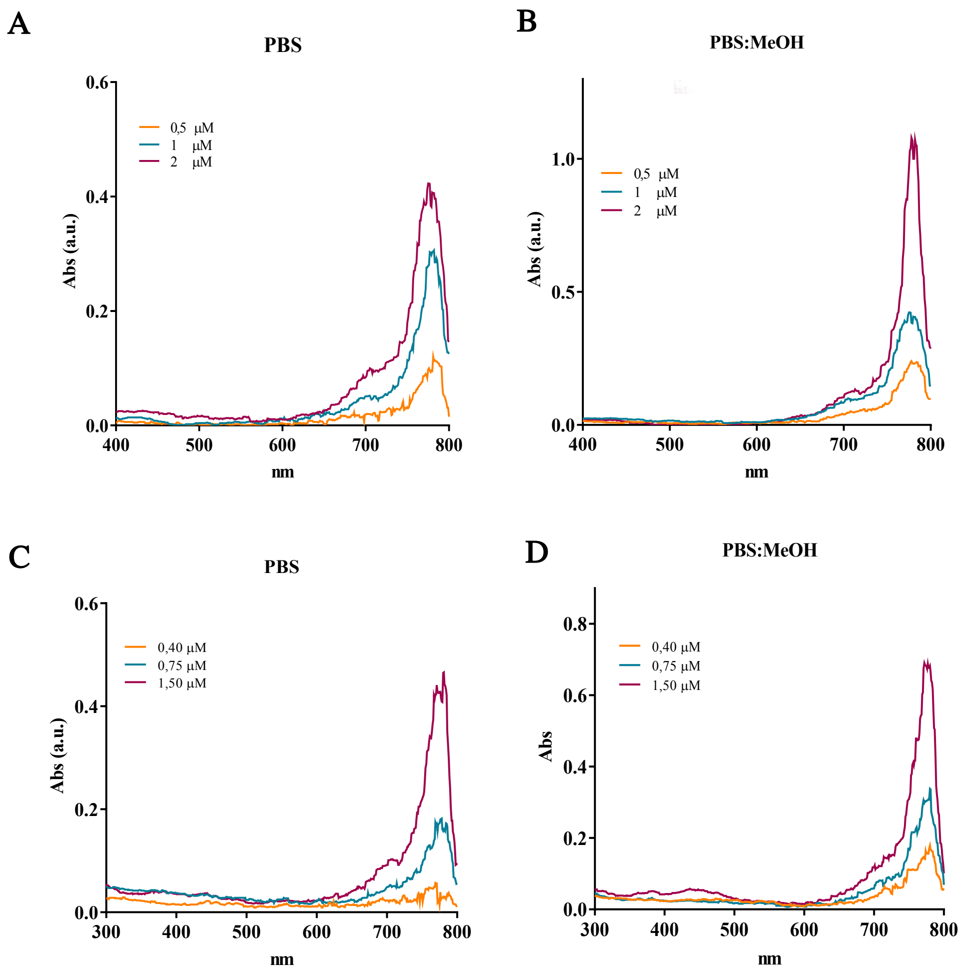

Figure S3 **(A)** Absorbance spectra of FAPI-IRDye800CW in PBS pH=7.4. **(B)** Absorbance spectra of FAPI-IRDye800CW in PBS:MeOH (1:1). **(C)** Absorbance spectra of FAPI-FNIRTag in PBS pH=7.4. **(D)** Absorbance spectra of FAPI-FNIRTag in PBS:MeOH (1:1).

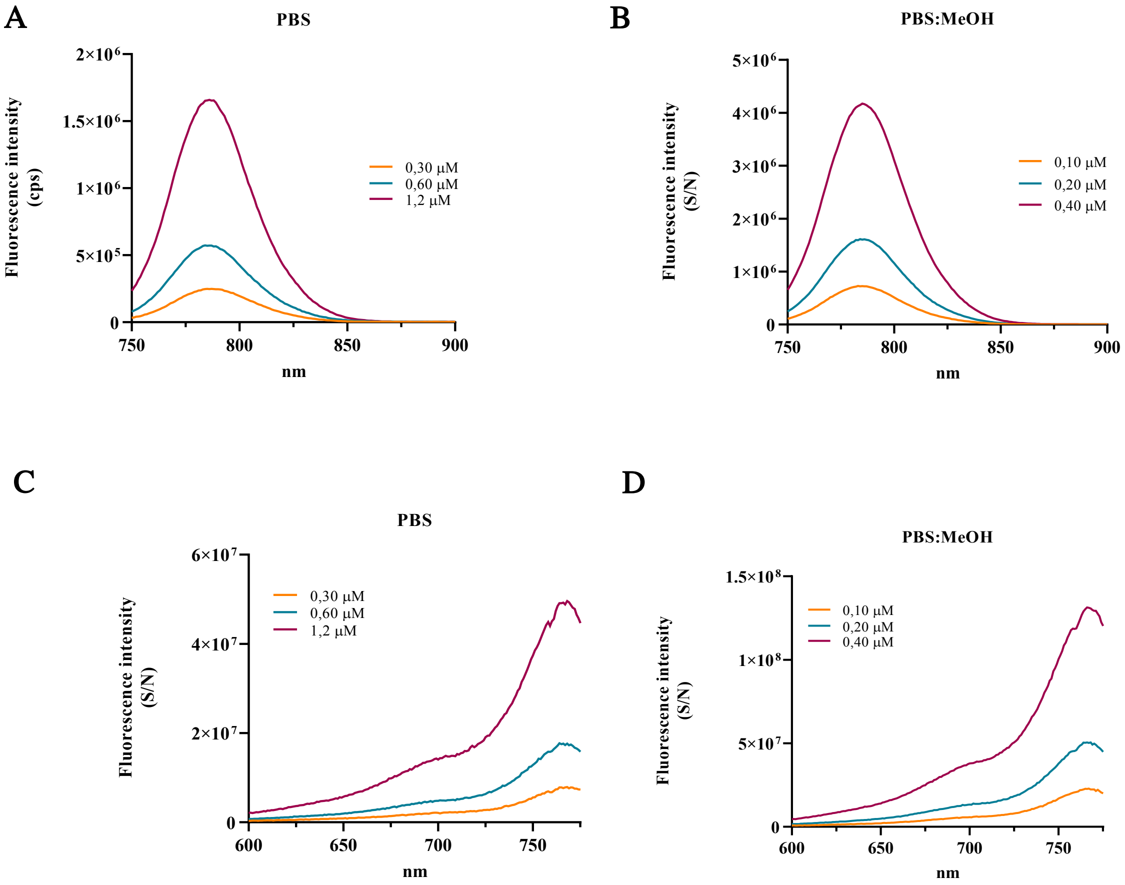

Figure S4 **(A)** Fluorescence emission spectra of FAPI-FNIRTag in PBS pH=7.4. **(B)** Fluorescence emission spectra of FAPI-FNIRTag in PBS:MeOH (1:1). **(C)** Fluorescence excitation spectra of FAPI-FNIRTag in PBS pH=7.4. **(D)** Fluorescence excitation spectra of FAPI-FNIRTag in PBS:MeOH.

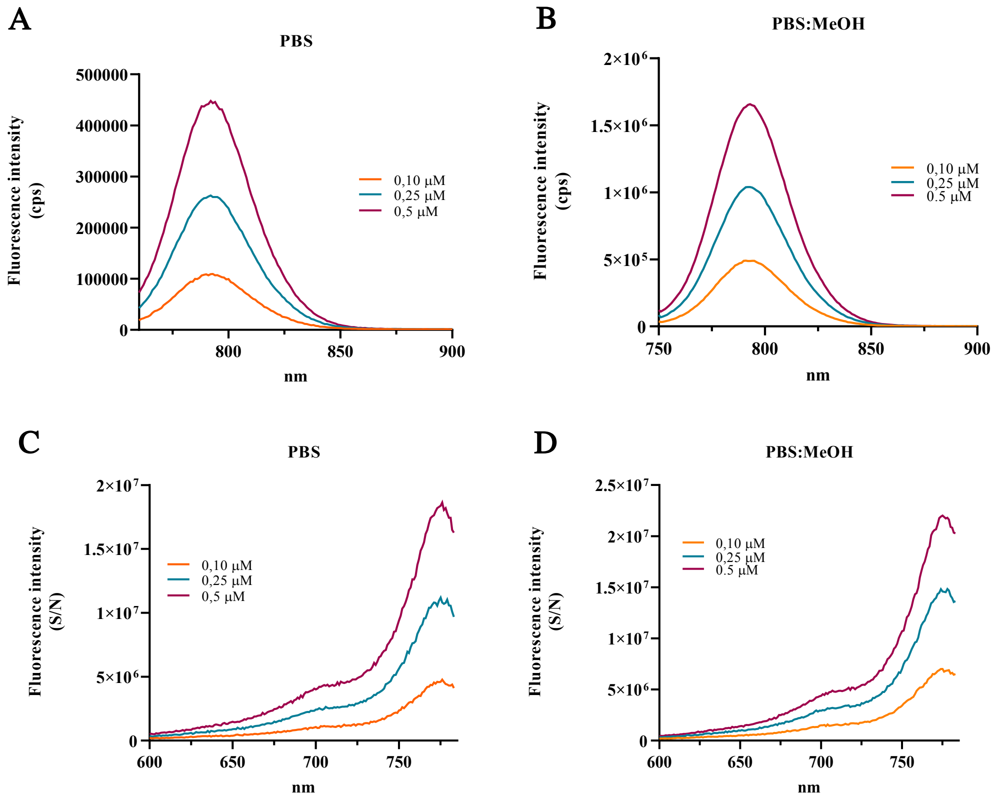

Figure S5 (A) Fluorescence emission spectra of FAPI-IRDye800CW in PBS pH=7.4. **(B)** Fluorescence emission spectra of FAPI-IRDye800CW in PBS:MeOH (1:1). **(C)** Fluorescence excitation spectra of FAPI-IRDye800CW in PBS pH=7.4. **(D)** Fluorescence excitation spectra of FAPI-IRDye800CW in PBS:MeOH.

Serum stability

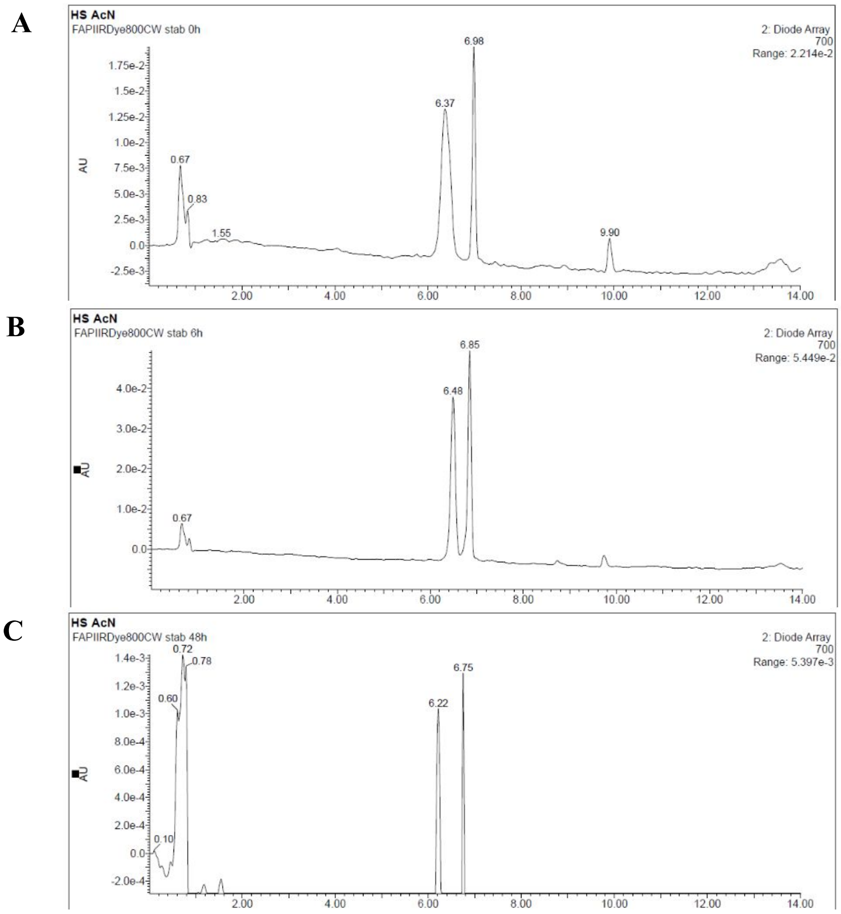

Figure S6 UPLC-UV/Vis spectrum at 700 nm of FAPI-IRDye800CW after 0, 6 and 48h incubation with human serum at 37˚C. MS analysis of peak at lower retention time revealed the same mass pattern of product, hence no degradation was found. It is possible to attribute this peak to a matrix effect of the human serum on retention process (ions, pH).

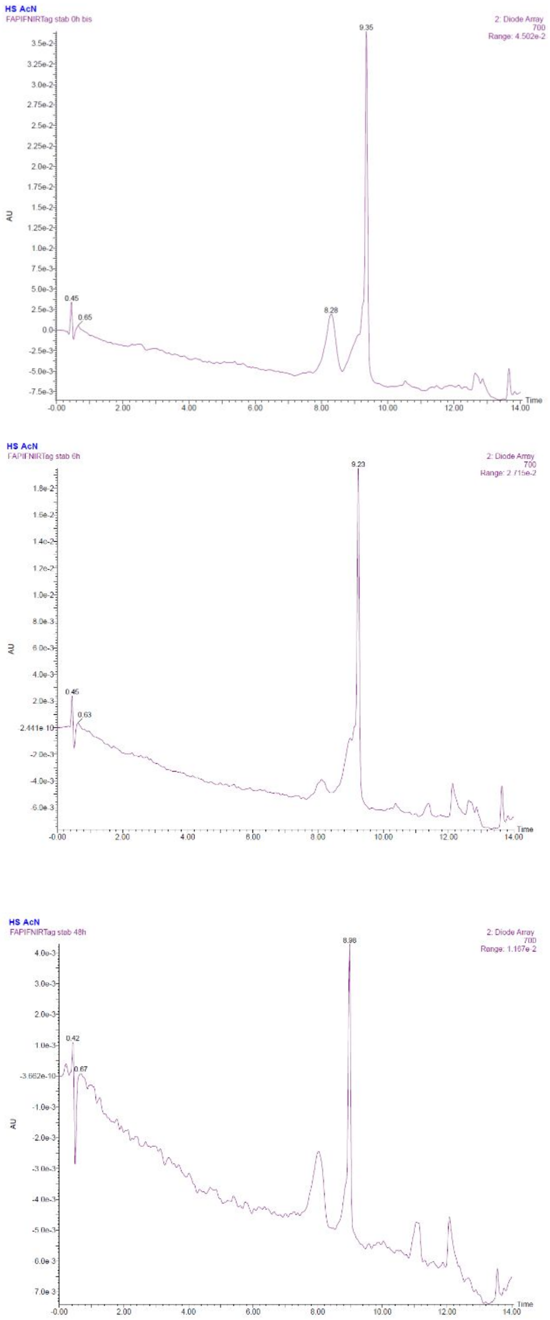

Figure S7 UPLC-UV/Vis spectrum at 700 nm of FAPI-FNIRTag after 0, 6 and 48h incubation with human serum at 37°C. MS analysis of peak at lower retention time revealed the same mass pattern of product, hence no degradation was found. It is possible to attribute this peak to a matrix effect of the human serum on retention process (ions, pH).

FAP expression quantification

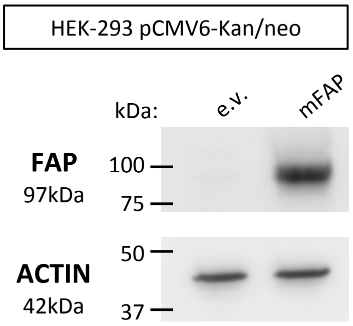

Figure S8 FAP expression quantification by WB analysis.

Preclinical model identification

Tumor digestion and flow cytometry

Anesthetized mice were sacrificed at day 35 through cervical dislocation. Tumor tissues were excised and divided into two parts to perform both flow cytometry analysis and immunohistochemical staining. For flow cytometry analysis, mushed tumor tissues were digested in DMEM added with dispase 3mg/ml (D4693 – Sigma Aldrich) and collagenase 1mg/ml (C0130 – Sigma Aldrich). Suspension was vortexed in orbital shaker for 30 minutes at 37˚C, filtered with cell strainer (70 μm) and centrifuged (10 minutes, 1400 rpm). $1*10^6$ cells were pelleted for each condition tested. Incubation with anti-FAP primary antibody (1h at 4˚C), anti-CD45 (30 min 4˚C), anti-F4/80 (30 min 4˚C) was performed. After washing with PBS, secondary antibody was incubated for 30 minutes at 4˚C.

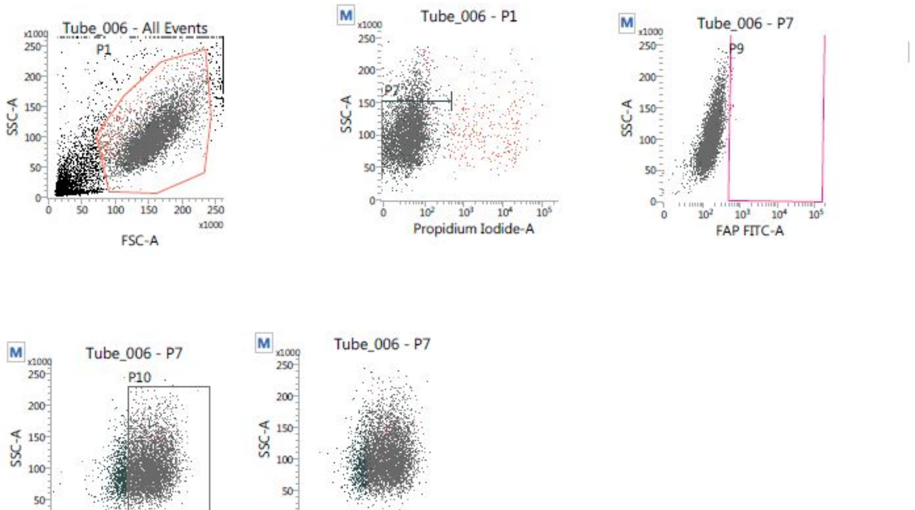

Tube_006 RunPointerStatistics

Name	Events	% Total	% Parent	FAP FITC-A Mean	Propidium Iodide-A Mean	F480 PE-Vio770-A Mean	F480 PE-Vio770-H Mean	probe APC-Vio770-A Mean
All Events	10,000	100.00	***	142	1,358	-1,939	-335	4,157
P1	6,034	60.34	60.34	179	640	-739	-114	5,139
P7	5,718	57.18	94.76	185	46	139	78	5,172
P9	21	0.21	0.37	630	134	235	220	7,262
P10	4,874	48.74	85.24	190	48	143	81	5,957

Figure S9 Flow cytometry results for FAPI-FNIRTag uptake on FAP-expressing cells (HEK293T-FAP) as dot plots.

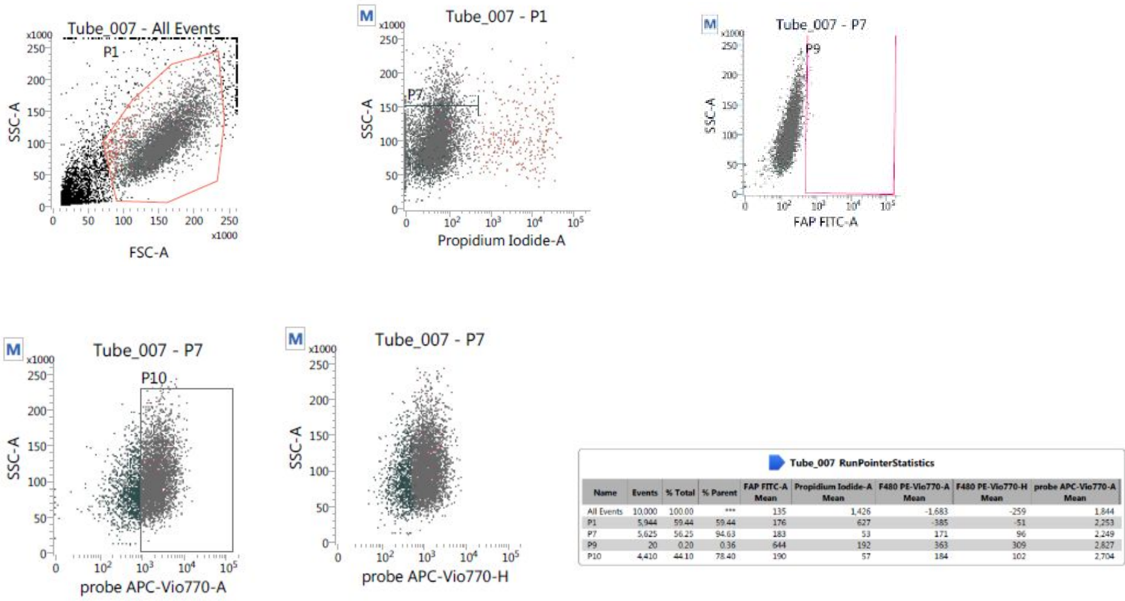

Tube_007 RunPointerStatistics

Name	Events	% Total	% Parent	FAP FITC-A Mean	Propidium Iodide-A Mean	F480 PE-Vio770-A Mean	F480 PE-Vio770-H Mean	probe APC-Vio770-A Mean
All Events	10,000	100.00	***	135	1,426	-1,683	-259	1,844
P1	5,944	59.44	59.44	176	627	-385	-51	2,253
P7	5,625	56.25	94.63	183	53	171	96	2,249
P9	20	0.20	0.36	644	192	363	309	2,827
P10	4,410	44.10	78.40	190	57	184	102	2,704

Figure S10 Flow cytometry results for FAPI-IRDye800CW uptake on FAP-expressing cells (HEK293T-FAP) as dot plots.
